# YTHDF3 Enhances Osteogenic Differentiation of Bone Marrow Mesenchymal Stem Cells in Osteoporosis by Promoting TBX19 Expression

**DOI:** 10.1111/cpr.70240

**Published:** 2026-06-03

**Authors:** Qianke Tao, Qiaonan Ye, Chengpeng Yang, Yuping Xie, Xuemei Long, Long Bai, Zhiyuan Zhang, Qianwei Li, Dan Tan, Jingang Xiao

**Affiliations:** ^1^ Department of Plastic and Burn Surgery The Affiliated Hospital, Southwest Medical University Luzhou China; ^2^ Luzhou Key Laboratory of Oral & Maxillofacial Reconstruction and Regeneration, The Affiliated Stomatological Hospital, Southwest Medical University Luzhou China; ^3^ Department of Oral and Maxillofacial Surgery The Affiliated Hospital, Southwest Medical University Luzhou China; ^4^ Department of Stomatology Leshan Municipal People's Hospital Leshan China; ^5^ Department of Oral Implantology The Affiliated Stomatological Hospital, Southwest Medical University Luzhou China; ^6^ Department of Periodontal and Mucosal Diseases The Affiliated Stomatological Hospital, Southwest Medical University Luzhou China

## Abstract

N^6^‐methyladenosine (m^6^A) modification is critically involved in regulating the osteogenic differentiation of bone marrow mesenchymal stem cells (BMSCs), yet the functional contributions of m^6^A reader proteins in osteoporosis remain poorly defined. In this study, we found that osteoporosis in rats correlates with severe bone loss and diminished osteogenic potential of BMSCs, accompanied by down‐regulation of the m^6^A reader YTHDF3. Functional assays showed that YTHDF3 promotes osteogenic differentiation of BMSCs. RNA‐sequencing analysis identified the transcription factor TBX19 as a key downstream mediator of YTHDF3. Subsequent investigations confirmed that *Tbx19* knockdown not only attenuated the osteogenic capacity of BMSCs but also abrogated the pro‐osteogenic effect of *Ythdf3* overexpression. Moreover, in vivo experiments demonstrated that *Ythdf3* overexpression enhances the bone‐forming ability of osteoporotic BMSCs. Collectively, our results reveal that YTHDF3 acts as a positive regulator of BMSC osteogenesis, largely through regulating TBX19, and that its down‐regulation contributes to osteoporotic pathogenesis. These findings propose YTHDF3 as a novel potential therapeutic target for the treatment of osteoporosis.

## Graphical abstract

In this study, OP‐BMSCs were successfully isolated from OVX osteoporotic rats. Mechanistic investigations revealed that the RNA‐binding protein YTHDF3 significantly upregulates *Tbx19* expression by enhancing the stability of *Tbx19* mRNA. Elevated *Tbx19* levels, in turn, activate key osteogenic molecules, effectively promoting the differentiation of OP‐BMSCs into osteoblasts and thereby enhancing their osteogenic capacity. In vivo experiments demonstrated that overexpression of *Ythdf3* markedly promoted new bone formation at local bone defect sites in osteoporotic rats, improved bone microarchitecture and accelerated the repair of osteoporotic bone defects.

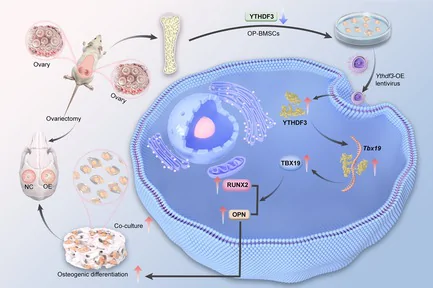

## 1 Introduction

The maintenance of bone homeostasis is a delicate dynamic equilibrium process that relies on the coordinated operation of bone formation and bone resorption [1]. An imbalance in this process is a primary pathological basis for metabolic bone diseases such as osteoporosis (OP), characterised by reduced bone mass, deterioration of bone microarchitecture and increased bone fragility [2, 3]. Bone marrow mesenchymal stem cells (BMSCs), as the primary precursor cells of osteoblasts, play a crucial role in bone formation and repair through their osteogenic differentiation capacity [4, 5, 6]. However, under osteoporotic conditions, particularly in postmenopausal osteoporosis (PMOP), the osteogenic differentiation potential of BMSCs is significantly impaired. This is accompanied by an enhanced tendency to differentiate into adipocytes, leading to reduced bone mass and accumulation of bone marrow adipose tissue, which has emerged as one of the core pathological mechanisms of OP [7, 8].

The differentiation fate of BMSCs is precisely regulated by a variety of signalling pathways and transcription factors. Key transcription factors such as RUNX2 and Osterix (*Sp7*) play pivotal roles in the osteogenic differentiation process [9, 10]. Furthermore, bone morphogenetic proteins (BMPs), particularly BMP2, along with the Wnt/β‐catenin signalling pathway, have been well‐established as classical pathways regulating the osteogenic differentiation of BMSCs [11, 12, 13]. In recent years, an increasing number of substances including individual Chinese herbal components, compounds and vesicles have been demonstrated to promote the osteogenic differentiation of BMSCs [14, 15, 16]. Although these substances are widely recognised for promoting bone formation, the mechanisms regulating the osteogenic differentiation of BMSCs remain unclear. Elucidating this intricate regulatory network is crucial for developing novel therapeutic strategies for bone diseases such as OP.

In recent years, the role of RNA epigenetic modifications, particularly N6‐methyladenosine (m^6^A), in the regulation of gene expression has attracted increasing attention. As the most prevalent internal chemical modification in eukaryotic mRNA, m^6^A is dynamically regulated by *writers*, *erasers* and *readers*, which collectively modulate RNA stability, splicing, localisation and translation efficiency [17, 18]. Although m^6^A has been widely implicated in various biological processes, including the differentiation and proliferation of BMSCs, its precise role and underlying mechanisms in the osteogenic differentiation of BMSCs remain to be fully elucidated [19, 20, 21].

YTHDF3, a crucial m^6^A reader protein, has been demonstrated to regulate the fate of target genes by recognising m^6^A modifications in various biological processes and diseases [22, 23, 24]. Notably, its role in bone metabolism is increasingly being uncovered. Research on ankylosing spondylitis (AS) has revealed that YTHDF3 expression is significantly upregulated in BMSCs of AS patients. It promotes the osteogenic differentiation and pathological ossification of BMSCs by recognising m^6^A modification sites on IL32 mRNA and enhancing its stability [25]. However, within the context of OP, the specific expression profile and functional role of YTHDF3 in BMSCs, along with its downstream effector molecule, remain to be clearly elucidated.

This study aims to investigate the regulatory role of YTHDF3 in the osteogenic differentiation of BMSCs and its underlying mechanisms within the pathological microenvironment of OP. We observed a downregulation of YTHDF3 expression in BMSCs derived from osteoporotic conditions. Subsequently, through lentivirus‐mediated overexpression and siRNA‐mediated knockdown approaches, we demonstrated that YTHDF3 is a key regulator promoting osteogenic differentiation of BMSCs. Transcriptome sequencing analysis suggested that TBX19 may act as a critical downstream effector of YTHDF3, a hypothesis that was further validated by rescue experiments involving siRNA‐mediated knockdown of *Tbx19*. In addition, in vivo studies using a rat calvarial defect model under osteoporotic conditions confirmed the potent pro‐osteogenic function of YTHDF3 within the osteoporotic microenvironment. These findings validate the regulatory function of the YTHDF3‐TBX19 axis in BMSCs osteogenesis, which not only reveals a novel role of m^6^A modification in OP but also provides a potential therapeutic target for YTHDF3‐based treatment of OP.

## 2 Materials and Methods

### 2.1 Animal Models

This research received approval from the Ethics Committee of Southwest Medical University (Ethics No. 20230703‐016) and was conducted in strict compliance with the ‘Guidelines for the Management and Use of Experimental Animals’ (2011) promulgated by the Ministry of Science and Technology of China. A cohort of 40 female Sprague–Dawley (SD) rats, aged 8 weeks, was procured from Southwest Medical University. Anaesthesia was initially induced through isoflurane inhalation and subsequently maintained via intraperitoneal administration of 2% pentobarbital sodium at a dosage of 30 mg/kg. To establish an OP model, bilateral ovariectomy (OVX) was performed on the SD rats. The sham‐operated control group underwent identical surgical procedures, excluding the removal of ovaries. Three months following the ovariectomy, femoral bone samples were collected from both the OVX and sham‐operated control groups. These samples were subjected to micro‐computed tomography (micro‐CT) scanning using a SCANCO Medical system. Post‐decalcification, the bone specimens were processed for histological analysis through Haematoxylin and Eosin (H&E) staining and Masson's trichrome staining [26].

### 2.2 Micro‐CT Analysis and Bone Tissue Section Staining

The femur samples were scanned using the SCANCO μCT 50 scanner (SCANCO Medical AG), and the data on trabecular bone within a 2 mm range at a distance of 1.5 mm from the growth plate were analysed. In addition, calvarial specimens with critical‐sized defects were also scanned using the same μCT system under identical scanning parameters. A cylindrical volume of interest (VOI) centred on the defect site was defined for quantitative analysis of new bone formation [27, 28]. The femur and calvarial samples were decalcified for 4 weeks, dehydrated, and then embedded in paraffin before being cut into 3‐μm‐thick sections. These sections were used for H&E staining and Masson's trichrome staining to assess tissue morphology and structure. In addition, immunohistochemical analysis was performed on the sections using the corresponding antibodies to evaluate the expression levels of specific proteins in the bone tissue of different groups of rats.

### 2.3 Isolation and Characterisation of BMSCs

BMSCs were isolated from SD rats using the whole bone marrow adherence method. Rats were euthanised by cervical dislocation, bilateral femurs/tibiae were aseptically excised. Marrow cavities were flushed with PBS, filtered through a 70‐μm strainer and centrifuged (1000 rpm, 5 min). The pellet was resuspended in α‐MEM supplemented with 10% FBS and 1% penicillin/streptomycin, seeded into T75 flasks at 1 × 10^6^ cells/mL, and cultured at 37°C/5% CO_2_. Non‐adherent cells were removed by medium replacement at 48 h, with subsequent changes every 3 days until 80%–90% confluency (7–10 days). Adherent cells were trypsinised (0.25% trypsin–EDTA, 1 min) and passaged at a 1:3 ratio; P3–P5 cells were used for characterisation. Identification included: (1) Morphology: homogeneous spindle‐shaped cells; (2) Flow cytometry: CD90/CD29/CD44 positivity > 95%, CD45/CD34/CD31 negativity < 5%; (3) Trilineage differentiation: osteogenesis (Alizarin Red S staining, ARS), adipogenesis (Oil Red O staining, ORO) and chondrogenesis (Alcian Blue staining, AB) [29].

### 2.4 Osteogenic Differentiation

Osteogenic differentiation induction culture of BMSCs at P3 was performed using osteogenic induction medium (Pricella). The osteogenic induction medium was then replaced every 3 days. After 5 days of induction, Alkaline Phosphatase staining (ALP) was conducted, and RNA and proteins were extracted for subsequent experiments. After 21 days of induction, ARS was performed.

### 2.5 ALP and ARS Staining

The third generation BMSCs were seeded in 12‐well plates and cultured until the cell confluence reached 70%–80%. Then, they were treated with osteogenic induction medium (Pricella). ALP activity was detected using an ALP detection kit (Sangon Biotech) on the 3rd and 5th days of osteogenic induction. On the 21st day of osteogenic induction, calcium nodules were stained using an ARS staining solution (Cyagen).

### 2.6 RNA m^6^A Dot Blot Assays

To study the overall mRNA m^6^A levels, m^6^A dot blot assays were performed. Total RNA was extracted from cells using TRIzol reagent (Ambion), and the RNA concentrations of each experimental group were adjusted to be consistent. A total RNA sample of 2 μg was denatured at 95°C for 3 min. Subsequently, the denatured samples were quickly transferred to a nitrocellulose membrane and crosslinked under a 302 nm UV lamp for 6 min. After washing the membrane with PBS buffer for 5 min, it was blocked with skim milk. The membrane was then incubated overnight at 4°C with an m^6^A antibody solution. The next day, after washing with PBS, goat anti‐rabbit IgG‐HRP antibody was incubated at room temperature for 1 h, followed by another wash with PBS. The membrane was visualised using Pierce ECL protein blotting substrate (Thermo), and the dot sample signals were quantitatively analysed using ImageJ software.

### 2.7 siRNA Interference and Lentiviral Transduction

The siRNA and negative control (NC) were synthesised by OBIO Technology, with the corresponding sequences listed in Table 1. Prior to transfection, BMSCs were cultured in antibiotic‐free medium. Transfection was then performed using the riboFECT CP Transfection Kit according to the manufacturer's protocol, with 100 nM siRNA applied for 12 h, after which the medium was replaced with fresh culture medium. Subsequent experiments were conducted 72 h post‐transfection.

**TABLE 1:** qPCR primer sequences and siRNA sequences.

Genes	Primer sequence 5′‐3′
*Gapdh*‐F	GCAAGTTCAACGGCACAG
*Gapdh*‐R	GCCAGTAGACTCCACGACA
*Runx2*‐F	GAACCAAGAAGGCACAGAC
*Runx2*‐R	AATGCGCCCTAAATCACTG
*Opn*‐F	ACAGTATCCCGATGCCACAG
*Opn*‐R	TGACTCATGGCTGGTCTTCC
*Ythdf3*‐F	CCTCACCAAGTGCAGTCTCA
*Ythdf3*‐R	CAGCACTGGATGCACCTCTA
*Tbx19*‐F	GGAGGGAGAAGGGAGATCCTAC
*Tbx19*‐R	GACGAAGTCCAACAGAAGGG
*Tbx19‐465&500‐F*	GGGACTGGACCCTAATGCTA
*Tbx19‐465&500‐R*	ACCCATTCCCCGTTGACATAC
*Tbx19‐773‐F*	AGAATGGCAGGCGGATGTT
*Tbx19‐773‐R*	CAACAGAAGGGAGTACATAGCA
*Tbx19‐1201‐F*	TTTGCGAGTGTATCTGTCCCC
*Tbx19‐1201‐R*	AATACCAGGGGCTATGGGCA
*Ythdf3*‐siRNA‐Sense	GGACGUGUGUUUAUAAUUAAGTT
*Ythdf3*‐siRNA‐Antisense	CUUAAUUAUAAACACACGUCCTT
*Tbx19*‐siRNA‐Sense	GAUUCAAGGAAGUCACUAATT
*Tbx19*‐siRNA‐Antisense	UUAGUGACUUCCUUGAAUCTT
NC‐siRNA‐Sense	UUCUCCGAACGUGUCACGUTT
NC‐siRNA‐Antisense	ACGUGACACGUUCGGAGAATT

*Ythdf3* overexpression (*Ythdf3*‐OE) and control (*Ythdf3*‐NC) lentiviruses were constructed by Genechem. A gradient transfection assay was first conducted, with cell morphology and fluorescence intensity observed and recorded under a fluorescence microscope to determine the optimal multiplicity of infection (MOI). Subsequent experiments were performed using the selected MOI for transfection.

### 2.8 RNA Isolation and Quantitative Real‐Time PCR (RT‐qPCR)

Total RNA was extracted from cultured cells using TRIzol reagent (Invitrogen) according to the manufacturer's protocol. The total RNA concentration of each sample was determined using a NanoDrop 2000 spectrophotometer (Thermo Fisher). Equal amounts of RNA were reverse transcribed into cDNA using HiScript III RT SuperMix for qPCR (+gDNA wiper) (Vazyme). RT‐qPCR was performed on the CFX Connect real‐time fluorescence quantitative PCR system (Bio‐Rad) using ChamQ Universal SYBR qPCR Master Mix (Vazyme). The mRNA expression levels were normalised to the internal reference gene *Gapdh*, and the relative mRNA expression levels were calculated using the 2^−ΔΔCt^ method. The primer sequences are detailed in Table 1. The RT‐qPCR reaction programme consisted of an initial denaturation at 95°C for 5 min, followed by 40 cycles of amplification.

### 2.9 Protein Extract and Western Blot Assay

After collecting the samples, a cell lysis buffer containing protease and phosphatase inhibitors (Beyotime) was added to the RIPA buffer (Beyotime). The samples were lysed using an ultrasonic disruptor (30% amplitude, 5 s, five times), followed by additional lysis on ice for 30 min. After centrifugation at 12,000 × *g* for 5 min, the supernatant was collected. The protein concentration was determined using a BCA kit (Beyotime), and the proteins were denatured at 100°C for 10 min in sodium dodecyl sulphate‐polyacrylamide gel electrophoresis (SDS‐PAGE) sample buffer. Equal amounts of protein were separated by SDS‐PAGE and transferred to a polyvinylidene difluoride (PVDF) membrane. After blocking the membrane with a rapid blocking buffer (Beyotime), the membrane was incubated overnight at 4°C with the primary antibody under gentle agitation. Following a 1‐h incubation with the secondary antibody at room temperature, the protein bands were visualised using the SuperPico ECL chemiluminescent reagent kit (Vazyme) and analysed for signal intensity using ImageJ software (National Institutes of Health, USA). The relevant antibody information is listed in Table 2.

**TABLE 2:** Antibodies used in this study.

Antibodies	Company	Application	Dilution fold
m^6^A antibody	Synaptic Systems	Dot blot	1:1000
RUNX2	Abcam	WB; IF	1:1000; 1:200
OPN	Abmart	WB; IF	1:1000; 1:200
YTHDF3	Abcam	WB; IHC; IF	1:1000; 1:200; 1:200
TBX19	Leading‐Biology	WB	1:1000
GAPDH	Proteintech	WB	1:4000

### 2.10 RNA‐Sequencing

BMSCs from both the *Ythdf3*‐NC and *Ythdf3*‐OE groups were subjected to osteogenic induction for 5 days prior to RNA extraction. Total RNA was isolated from BMSCs using TRIzol Reagent according to the manufacturer's protocol. Following quality control, RNA purification, reverse transcription, library construction and sequencing were performed by Shanghai Majorbio Bio‐pharm Biotechnology Co. Ltd. (Shanghai, China). Stranded mRNA sequencing libraries were prepared using the Illumina Stranded mRNA Prep, Ligation Kit (San Diego, CA) with 1 μg of total RNA as input. Sequencing was carried out on the NovaSeq X Plus platform (PE150) with NovaSeq Reagent Kits. Differential expression analysis was conducted with DESeq2. Genes with |log_2_(fold change)| ≥ 1.5 and *p* < 0.05 were defined as significantly differentially expressed.

### 2.11 RNA Immunoprecipitation and qPCR (RIP‐qPCR)

RNA immunoprecipitation (RIP) assays were performed using the Magna RIP RNA‐Binding Protein Immunoprecipitation Kit (Millipore, USA) according to the manufacturer's instructions. Briefly, BMSCs were lysed, and the clarified cell lysates were incubated with magnetic beads conjugated to either anti‐YTHDF3 antibody (Abcam; dilution 1:50) or normal rabbit IgG (as a NC). After extensive washing, RNA was extracted from the immunoprecipitated complexes, reverse‐transcribed into cDNA and subjected to quantitative PCR (qPCR) for *Tbx19* mRNA detection.

### 2.12 m^6^A MeRIP‐qPCR Assays

m^6^A‐modified RNA fragments were enriched using the EpiQuik CUT&RUN m^6^A RNA Enrichment Kit (Epigentek) following the manufacturer's protocol. Briefly, total RNA was extracted and fragmented, then incubated with an anti‐m^6^A antibody (Synaptic Systems; dilution 1:100) together with Protein A/G magnetic beads. Normal IgG was used as a NC for immunoprecipitation. After stringent washing, bound RNA was eluted, purified, reverse‐transcribed into cDNA and analysed by quantitative PCR (qPCR). Putative m^6^A modification sites within *Tbx19* mRNA were predicted using the SRAMP online tool (http://www.cuilab.cn/sramp), and site‐specific primers are listed in Table 1.

### 2.13 RNA Stability Assays

RNA stability was evaluated by treating *Ythdf3*‐NC and *Ythdf3*‐OE cells with actinomycin D (ActD, 5 μg/mL) to block de novo transcription. Total RNA was isolated at 0, 2, 4, 6 and 8 h after ActD addition. The decay kinetics of *Tbx19* mRNA were determined by quantitative real‐time PCR (qPCR), and the relative mRNA levels were used to assess the impact of YTHDF3 overexpression on *Tbx19* transcript stability.

### 2.14 Implantation of BMSC‐Seeded Biphasic Calcium Phosphate (BCP) into Bilateral Calvarial Defects of OP Rats

To investigate the regulatory role of YTHDF3 in bone defect repair under osteoporotic conditions, we employed a calvarial critical‐sized defect model, as this approach has been well established for evaluating bone regeneration in vivo [30, 31]. *Ythdf3*‐OE and *Ythdf3*‐NC groups (1 × 10^6^ cells per group) were co‐cultured with BCP scaffolds in 24‐well plates for 3 days to allow cell adhesion and proliferation. After anaesthesia, OP rats were fixed in a prone position, followed by skin preparation and disinfection of the calvarial region. Bilateral 5‐mm critical‐sized calvarial defects were created using a trephine drill under sterile conditions. The cell‐seeded BCP scaffolds were implanted into the defects, with the left side receiving NC‐group scaffolds and the right side receiving *Ythdf3*‐OE‐group scaffolds. Subsequently, at 8 and 12 weeks after implantation, we harvested the calvarial specimens for follow‐up experiments such as micro‐CT scanning and H&E staining [32].

### 2.15 Quantification and Statistical Analysis

Statistical analysis was performed using GraphPad Prism (version 9.1.1, GraphPad Software). The distribution characteristics of the data were determined using the Shapiro–Wilk normality test. Data that followed a normal distribution were expressed as mean ± standard deviation. Comparisons between two groups were conducted using unpaired or paired two‐tailed Student's *t*‐test, while comparisons among multiple groups were conducted using one‐way analysis of variance (ANOVA) followed by Tukey's multiple comparisons test. Non‐normally distributed data were expressed as median and interquartile range, and analysed using the Mann–Whitney *U* test or Kruskal–Wallis test. *p* < 0.05 was considered statistically significant. The specific number of biological replicates (*n*) and details of the statistical analysis are provided in the figure legend.

## 3 Results

### 3.1 Bone Loss and Impaired Osteogenic Differentiation Ability are Presented in BMSCs of OP Rats

OP rat models were established via bilateral OVX. Micro‐CT analysis of femurs at 3 months post‐surgery revealed significant reductions in trabecular bone volume fraction (Tb.BV/TV) and trabecular number (Tb.N), along with increased trabecular spacing (Tb.Sp) in the OVX group compared to the sham‐operated group, confirming the successful establishment of OP models (Figure 1A,B). Subsequent histological examination of femurs (H&E staining and Masson staining) likewise validated the osteoporotic phenotype characterised by bone loss in OP rats (Figure 1C,D).

**FIGURE 1:**
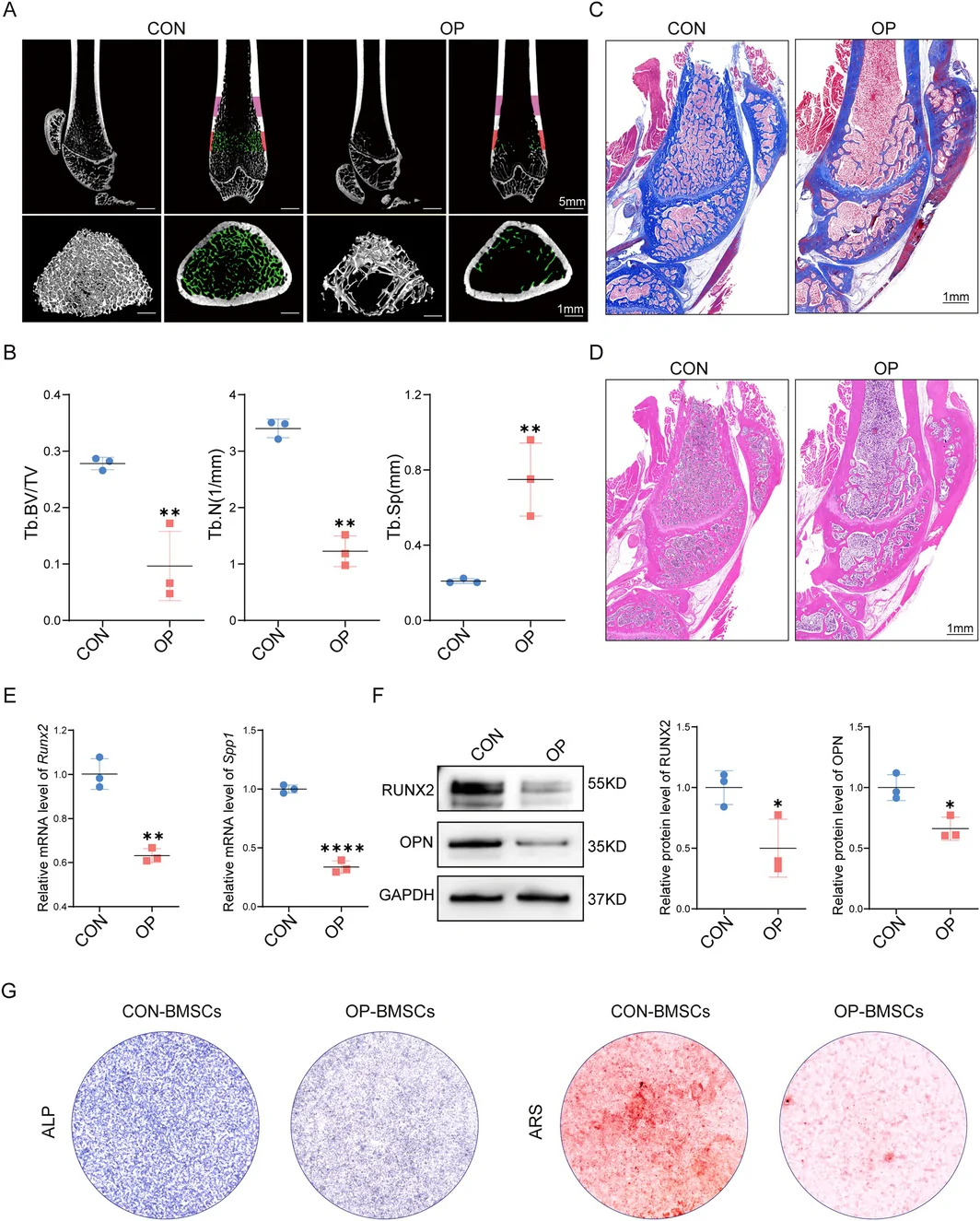
Reduction of the osteogenic differentiation potential of bone marrow mesenchymal stem cells in osteoporosis. (A) Representative micro‐CT images of the femoral trabecular bone. (B) Statistical analysis of the trabecular bone parameters. (C, D) Representative images of H&E and Masson staining of the femur. (E, F) RT‐qPCR and Western blot analysis of the mRNA and protein expression levels of the osteogenesis‐related genes *Runx2* and *Spp1*. *Gapdh* was used for normalisation. (G) Representative images of ALP staining (Day 7) and ARS staining (Day 21). Performed to compare the early (ALP activity) and late (mineralisation) stages of osteogenic differentiation between the two groups. All data are presented as means ± SD from three independent experiments (*n* = 3). *p < 0.05, **p < 0.01, ****p < 0.0001.

Primary BMSCs were isolated and cultured from femoral bone marrow using the whole bone marrow adherent method. Under microscopic observation, the primary BMSCs exhibited adherent growth with a spindle‐shaped, plump and evenly distributed morphology (Figure S1A). Flow cytometric analysis confirmed positive expression of mesenchymal markers (CD29, CD44 and CD90) alongside negative expression of haematopoietic lineage markers (CD34, CD45) and endothelial cell marker (CD31), indicating the mesenchymal origin (Figure S1B). In vitro ARS, ORO and AB differentiation assays confirmed its tri‐lineage differentiation potential (Figure S1C).

We cultured control group BMSCs (CON‐BMSCs) and osteoporosis group BMSCs (OP‐BMSCs) in osteogenic induction medium respectively to evaluate the differences in their osteogenic capabilities. After osteogenic induction, RNA and protein were extracted for examination. It was found that both the transcriptional and protein levels of osteogenesis‐related genes *Runx2* and *Spp1* were decreased in OP‐BMSCs (Figure 1E,F). ALP staining showed that the activity of ALP was significantly reduced in OP‐BMSCs. ARS results indicated that mineralised nodules were formed in both groups, but the number of nodules formed by OP‐BMSCs was significantly lower than that by CON‐BMSCs (Figure 1G). These results confirm that the osteogenic differentiation capacity of OP‐BMSCs is impaired, which is considered one of the important reasons for bone loss in OP rats.

### 3.2 YTHDF3 Expression is Suppressed under Osteoporotic Conditions

To investigate the role of YTHDF3 in the osteogenic differentiation of BMSCs and the pathogenesis of OP, we assessed YTHDF3 expression in femurs via immunohistochemistry. The results revealed a significant reduction in YTHDF3 expression within the bone marrow of OP rats (Figure 2A). Dot blot analysis revealed a global reduction in m^6^A methylation levels in OP‐BMSCs (Figure 2B). Furthermore, both the transcriptional and protein levels of YTHDF3 were found to be significantly decreased in these cells (Figure 2C,D). These findings suggest that YTHDF3 may be implicated in the pathogenesis of OP.

**FIGURE 2:**
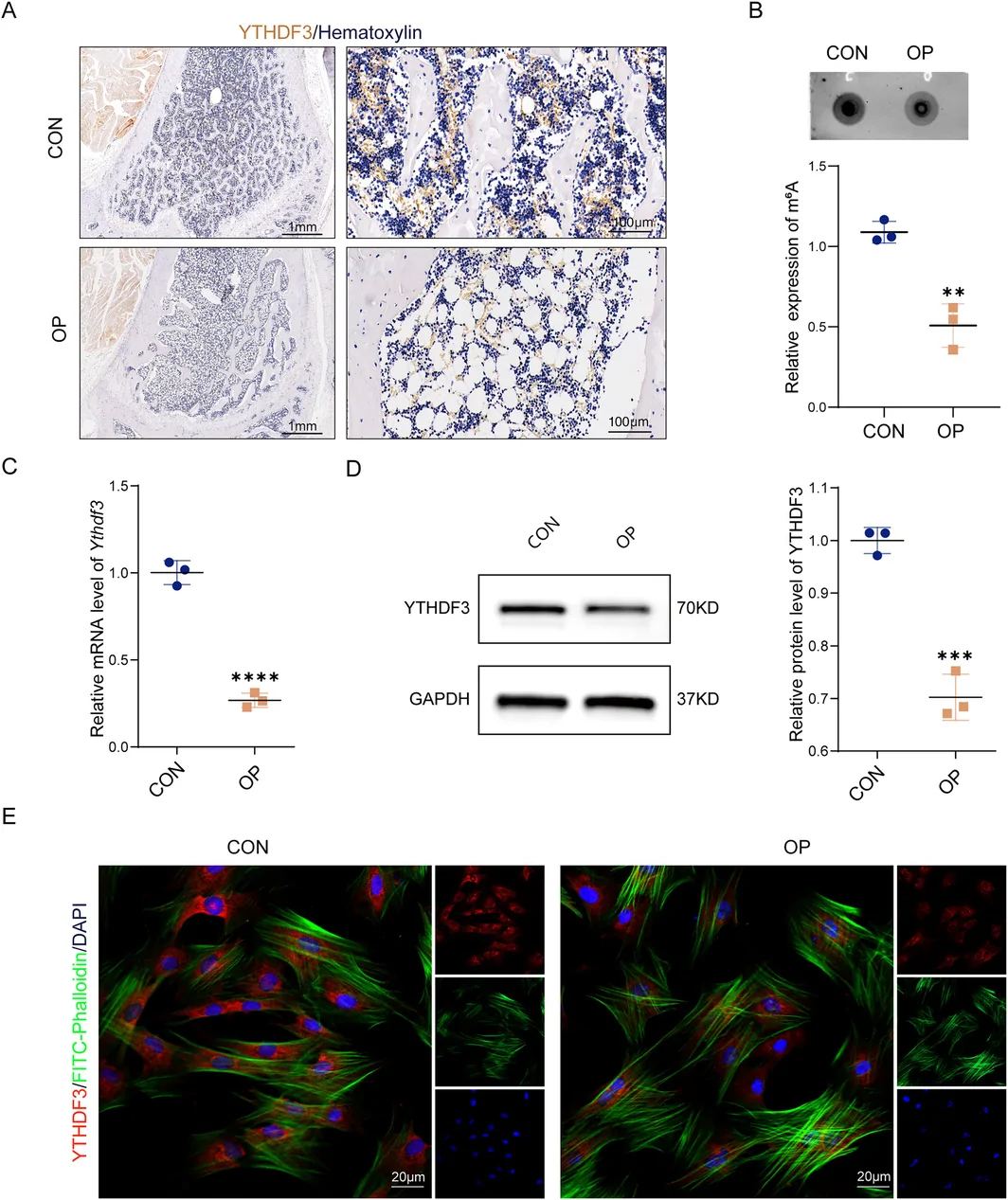
YTHDF3 expression is downregulated in the femur and BMSCs under osteoporotic conditions. (A) Representative immunohistochemical images of YTHDF3 expression in the femur from CON and OP group rats. Scale bar = 100 μm. (B) Dot blot analysis of m^6^A levels in CON‐BMSCs and OP‐BMSCs. (C, D) Analysis of *Ythdf3* transcriptional and protein expression by RT‐qPCR and Western blot using GAPDH for normalisation. (E) Representative immunofluorescence images of YTHDF3 in BMSCs from the CON and OP groups. Scale bar = 20 μm. All data are presented as means ± SD from three independent experiments (*n* = 3). **p < 0.01, ***p < 0.001, ****p < 0.0001.

### 3.3 YTHDF3 Promotes Osteogenic Differentiation of BMSCs

To determine the role of YTHDF3 in the osteogenic differentiation of BMSCs, we conducted loss‐of‐function and gain‐of‐function experiments in BMSCs. Overexpression of *Ythdf3* was achieved by infecting BMSCs with a *Ythdf3* overexpressing lentivirus. Seventy‐two hour post‐transfection, the culture medium was replaced with osteogenic induction medium for 5 days. Subsequent qRT‐PCR and Western blot analyses confirmed the upregulation of YTHDF3 at both the mRNA and protein levels. Concurrently, the mRNA and protein levels of osteogenic marker genes, such as *Runx2* and *Spp1*, were also significantly increased compared to the control group (Figure 3A–C). Immunofluorescence staining further verified the upregulation of RUNX2 and OPN following *Ythdf3* overexpression (Figure 3D). ALP and ARS staining were performed on BMSCs after 7 and 21 days of osteogenic induction, respectively. The *Ythdf3* overexpression group exhibited enhanced ALP activity and a greater number of mineralised nodules (Figure 3E). Collectively, these findings demonstrate that *Ythdf3* overexpression significantly promotes the osteogenic potential of BMSCs.

**FIGURE 3:**
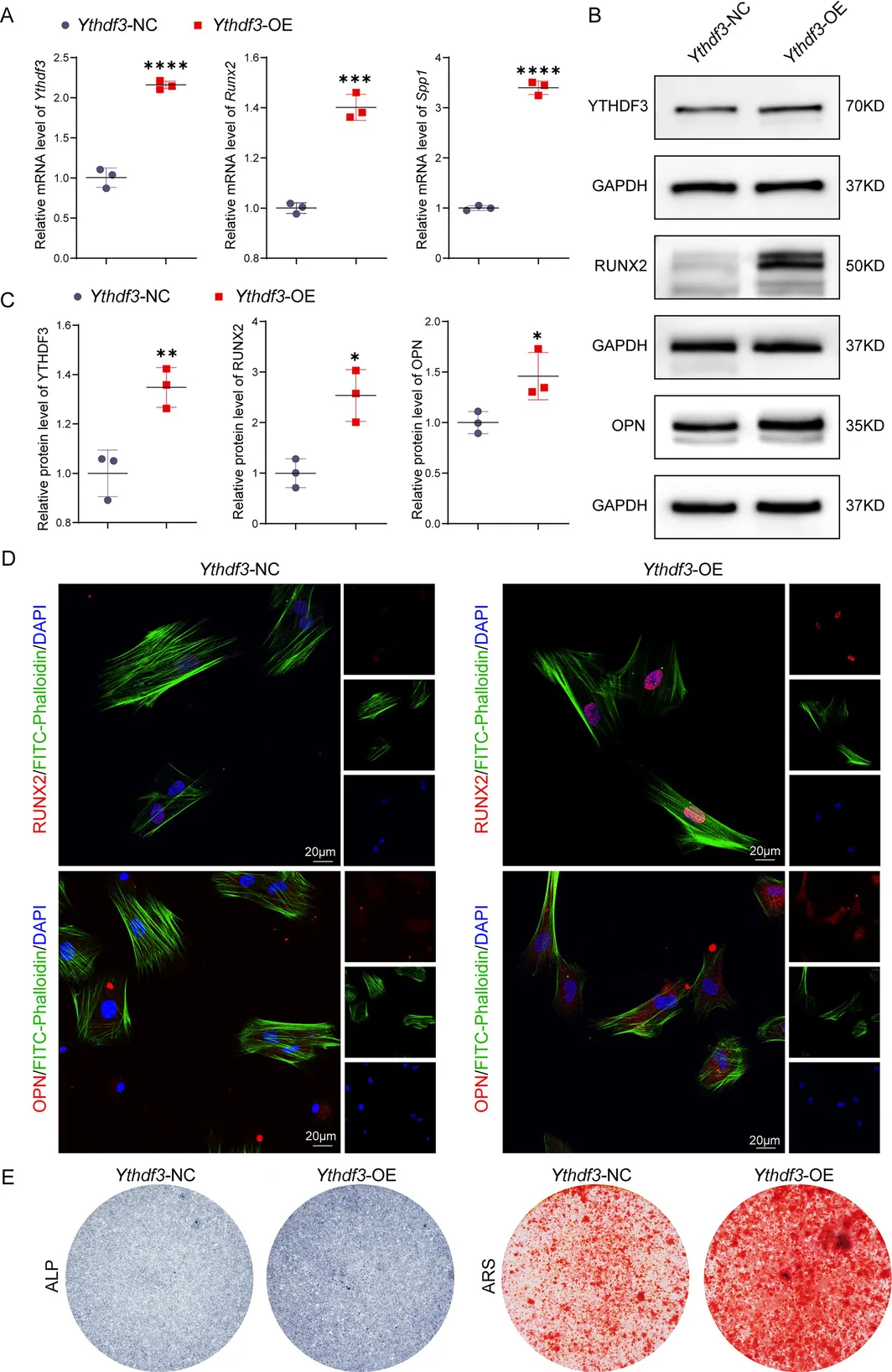
*Ythdf3* overexpression enhances the osteogenic potential of BMSCs. (A) mRNA levels of *Ythdf3*, *Runx2*, and *Spp1* were measured by RT‐qPCR with *Gapdh* normalisation. (B, C) Protein levels of YTHDF3, RUNX2, and OPN were assessed by Western blot using GAPDH as a loading control. (D) Representative immunofluorescence images of RUNX2 and OPN in BMSCs from the NC and *Ythdf3*‐OE groups. Scale bar = 20 μm. (E) Representative images of ALP staining (Day 7) and ARS staining (Day 21). All data are presented as means ± SD from three independent experiments (*n* = 3). *p < 0.05, **p < 0.01, ***p < 0.001, ****p < 0.0001.

*Ythdf3* was knocked down by transfecting cells with siRNA (*Ythdf3*‐siRNA). Following osteogenic induction for 5 days, RT‐qPCR and Western blot analyses consistently demonstrated that silencing *Ythdf3* significantly downregulated the transcriptional and translational expression levels of the key osteogenic marker genes, *Runx2* and *Spp1* (Figure 4A–C). The inhibition of RUNX2 and OPN expression following *Ythdf3* knockdown was further confirmed by immunofluorescence staining (Figure 4D). Consistent with the downregulation of osteogenic markers, *Ythdf3* knockdown functionally impaired osteogenic differentiation of BMSCs, as shown by decreased ALP activity and reduced matrix mineralisation (Figure 4E). In summary, knocking down *Ythdf3* dampens the osteogenic potential of BMSCs.

**FIGURE 4:**
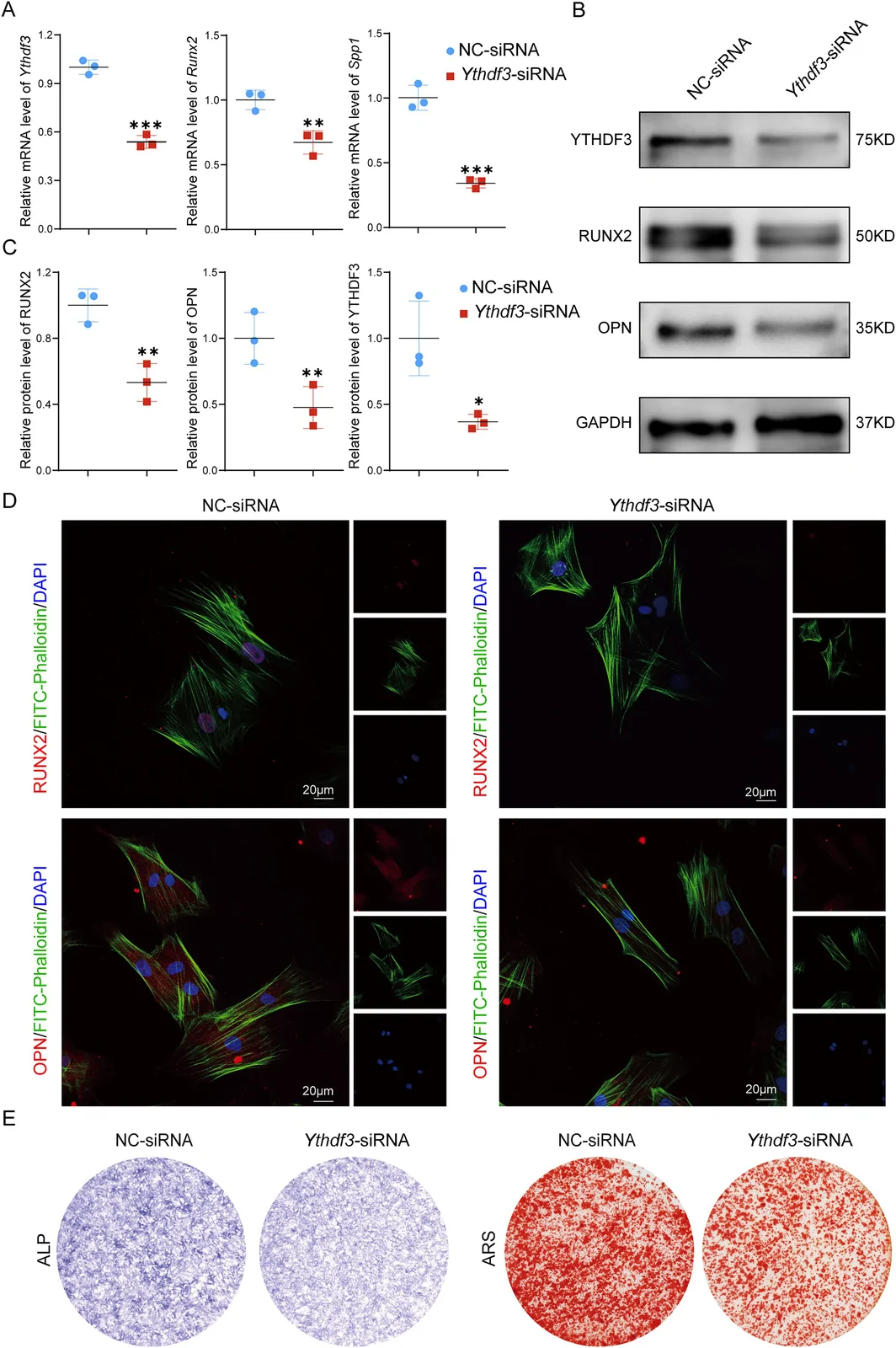
*Ythdf3* knockdown impairs the osteogenic potential of BMSCs. (A) mRNA levels of *Ythdf3*, *Runx2*, and *Spp1* were measured by RT‐qPCR with *Gapdh* normalisation. (B, C) Protein levels of YTHDF3, RUNX2, and OPN were assessed by Western blot using GAPDH as a loading control. (D) Representative immunofluorescence images of RUNX2 and OPN in BMSCs from the NC and *Ythdf3*‐OE groups. Scale bar = 20 μm. (E) Representative images of ALP staining (Day 7) and ARS staining (Day 21). All data are presented as means ± SD from three independent experiments (*n* = 3). *p < 0.05, **p < 0.01, ***p < 0.001.

Both loss‐of‐function and gain‐of‐function studies in BMSCs demonstrate that YTHDF3 plays a crucial role in promoting the osteogenic differentiation of BMSCs.

### 3.4 RNA‐Sequencing Indicates *Tbx19* may be a Critical Downstream Effector of *Ythdf3*

While previous studies have established the pro‐osteogenic role of YTHDF3 in BMSCs, its precise molecular mechanisms remain incompletely understood. To elucidate these mechanisms, we performed RNA‐sequencing on *Ythdf3*‐OE and *Ythdf3*‐NC groups. Bioinformatic analysis identified 469 significantly upregulated and 205 significantly downregulated genes, with a Circos heatmap visually confirming the pronounced transcriptomic differences induced by *Ythdf3* overexpression (Figure 5A,B). Gene Ontology (GO) annotation analysis of these differentially expressed genes (DEGs) revealed that *Ythdf3* overexpression broadly modulates diverse cellular processes, including metabolism, immune response and transcription factor activity (Figure 5C). Among the DEGs, *Tbx19* emerged as one of the most markedly altered. To determine the role of *Tbx19* in the osteogenic differentiation of BMSCs, we inhibited *Tbx19* expression using siRNA targeting *Tbx19* (*Tbx19*‐siRNA). Functional validation demonstrated that knockdown of *Tbx19* significantly suppressed both the transcriptional and translational expression of the key osteogenic markers *Runx2* and *Spp1* (Figure 5D–F). Collectively, these findings indicate that *Tbx19* may act as a critical downstream effector in the *Ythdf3*‐mediated osteogenic differentiation pathway. Based on the aforementioned results, we hypothesise that *Tbx19* may act as a critical downstream effector in the YTHDF3‐mediated osteogenic differentiation pathway of BMSCs.

**FIGURE 5:**
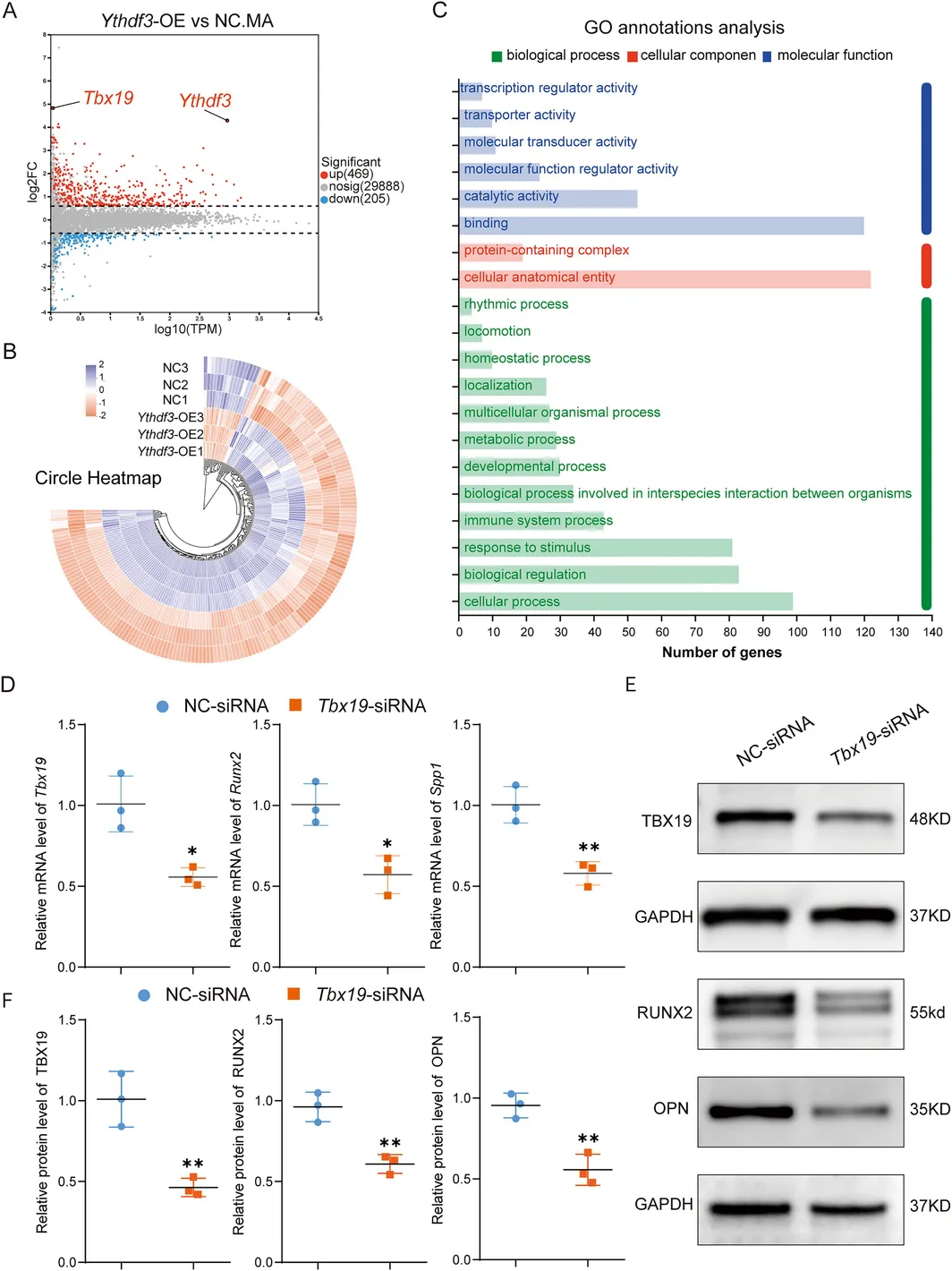
RNA‐seq identifies TBX19 as a key downstream effector of YTHDF3. (A) MA plot comparing RNA expression between *Ythdf3*‐OE and *Ythdf3*‐NC. (B) Circular heatmap displaying hierarchical clustering of differentially expressed genes across *Ythdf3*‐OE and *Ythdf3*‐NC. (C) Gene Ontology (GO) enrichment analysis of differentially expressed genes, categorised into biological processes, cellular components, and molecular functions. (D) mRNA levels of *Tbx19*, *Runx2*, and *Spp1* were measured by RT‐qPCR with *Gapdh* normalisation. (E, F) Protein levels of TBX19, RUNX2, and OPN were assessed by Western blot using GAPDH as a loading control. All data are presented as means ± SD from three independent experiments (*n* = 3). *p < 0.05, **p < 0.01.

### 3.5 Knockdown of *Tbx19* Suppresses Osteogenic Differentiation Potential of BMSCs and Reverses the Pro‐Osteogenic Effect of YTHDF3

To validate whether the pro‐osteogenic function of YTHDF3 is mediated through enhanced *Tbx19* expression, we knocked down *Tbx19* in BMSCs overexpressing *Ythdf3* (*Ythdf3‐*OE *+ Tbx19‐*siRNA). Consistent with our previous findings, qPCR and Western blot analyses demonstrated that overexpression of *Ythdf3* significantly enhanced the transcriptional and translational levels of *Runx2* and *Spp1*. And it can be observed that overexpression of *Ythdf3* significantly promoted *Tbx19* expression, consistent with our RNA‐sequencing analysis. Interestingly, these pro‐osteogenic effects were markedly attenuated upon *Tbx19* knockdown (Figure 6A–C). Consistently, the promotive effects of *Ythdf3* overexpression on ALP activity and matrix mineralisation, as assessed by ALP and ARS staining, were effectively reversed by *Tbx19* knockdown (Figure 6D). Collectively, these data indicate that YTHDF3 promotes osteogenic differentiation of BMSCs by regulating its downstream effector TBX19.

**FIGURE 6:**
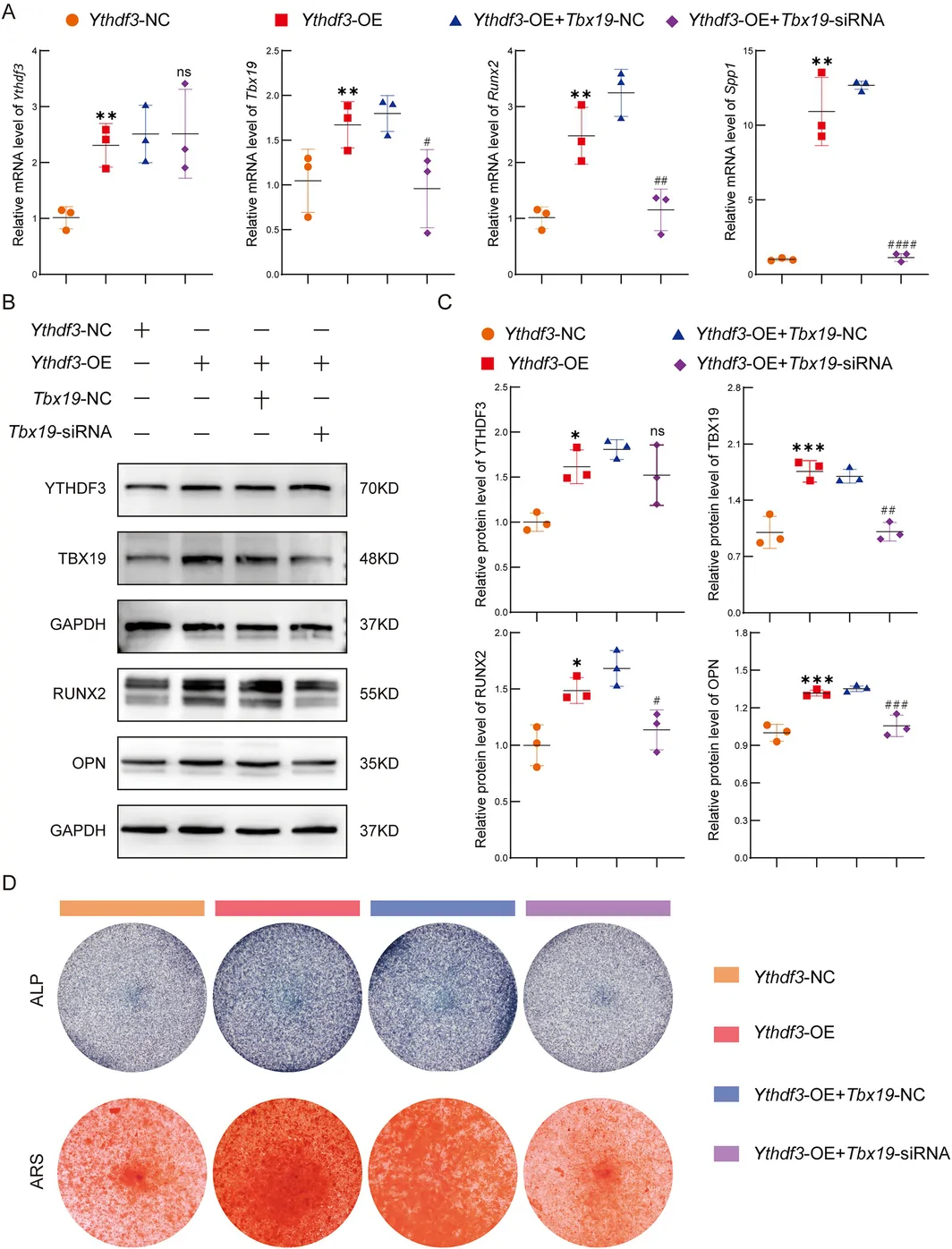
YTHDF3‐mediated osteogenic differentiation of BMSCs is dependent on TBX19 expression. (A) mRNA levels of *Ythdf3*, *Tbx19*, *Runx2*, and *Spp1* were measured by RT‐qPCR with *Gapdh* normalisation. (B, C) Protein levels of YTHDF3, TBX19, RUNX2, and OPN were assessed by Western blot using GAPDH as a loading control. (D) Representative images of ALP staining (day 7) and ARS staining (day 21). All data are presented as means ± SD from three independent experiments (*n* = 3). *p < 0.05, **p < 0.01, ***p < 0.001 versus *Ythdf3*‐NC samples; ^#^ *p* < 0.05, ^##^ *p* < 0.01, ^###^ *p* < 0.001, ^####^ *p* < 0.0001 versus *Ythdf3*‐OE+*Tbx19*‐NC; ns, not significant.

### 3.6 YTHDF3 Recognises m^**6**^A Modifications in the Coding Sequence (CDS) of *Tbx19* mRNA to Regulate Its Stability and Expression

To elucidate the molecular mechanism underlying the interaction between YTHDF3 and *Tbx19*, we first validated their physical association using RNA immunoprecipitation followed by quantitative PCR (RIP‐qPCR). Significant enrichment of *Tbx19* mRNA was detected in YTHDF3 immunoprecipitates (Figure 7A), confirming a direct molecular interaction. We further investigated whether YTHDF3 affects the stability of *Tbx19* mRNA. Upon transcriptional inhibition with actinomycin D (5 μg/mL), qPCR analysis revealed that overexpression of YTHDF3 significantly suppressed *Tbx19* mRNA degradation at multiple time points (Figure 7B), indicating that YTHDF3 stabilises *Tbx19* transcripts by inhibiting their decay.

**FIGURE 7:**
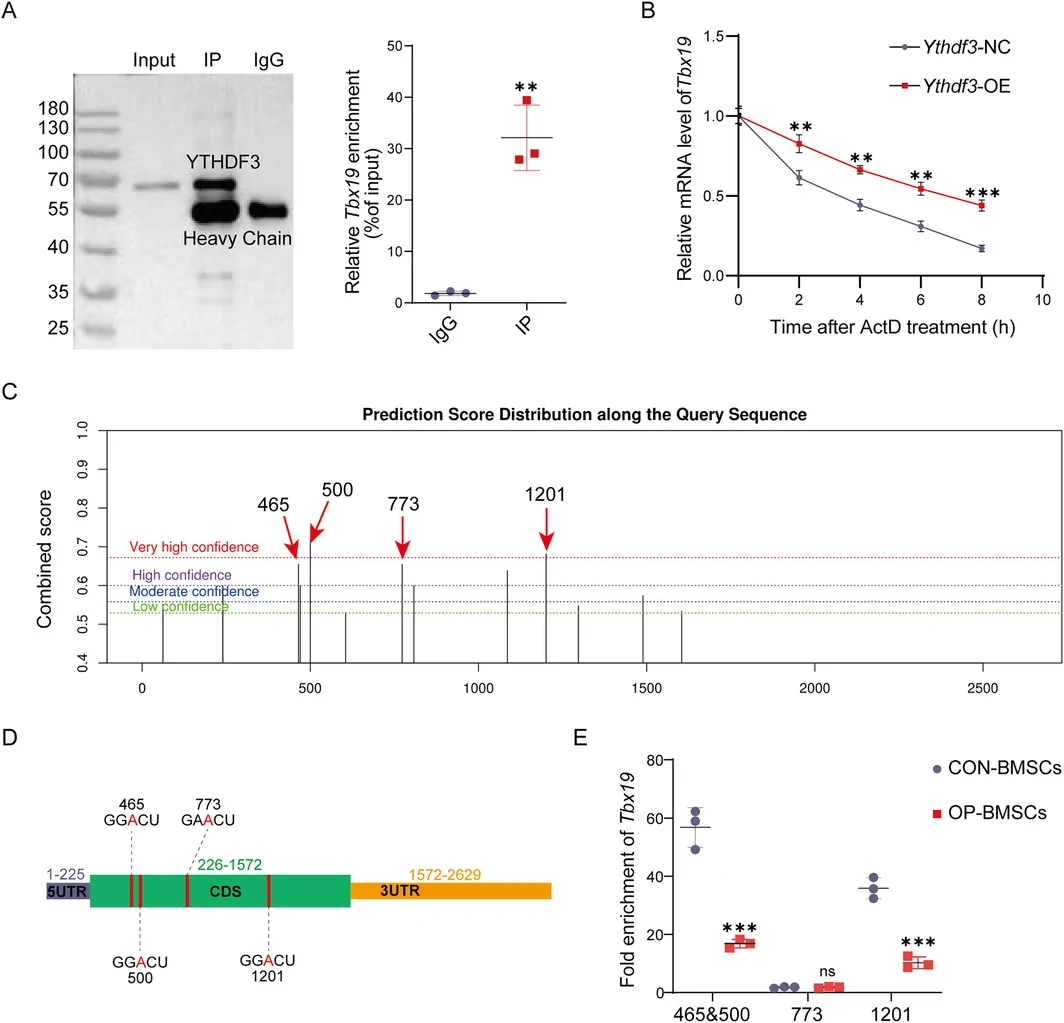
YTHDF3 binds to and stabilises *Tbx19* mRNA in an m^6^A‐dependent manner. (A) RIP‐qPCR analysis reveals significant enrichment of *Tbx19* mRNA in YTHDF3 immunoprecipitates compared with IgG control. (B) *Tbx19* mRNA decay kinetics after Actinomycin D treatment in *Ythdf3*‐NC and *Ythdf3*‐OE cells; relative mRNA levels normalised to time 0 over 8 h. (C) Schematic illustration of predicted m^6^A modification sites on *Tbx19* mRNA, as identified by SRAMP analysis. (D) Schematic representation of the *Tbx19* mRNA structure, indicating predicted m^6^A‐modified motifs at nucleotide positions 465, 500, 773, and 1201. (E) MeRIP‐qPCR analysis revealed that, compared with CON‐BMSCs, the m^6^A modification level at a specific site of the *Tbx19* gene was significantly reduced in OP‐BMSCs. All data are presented as means ± SD from three independent experiments (*n* = 3). **p < 0.01, ***p < 0.001, ns, not significant.

To identify potential regulatory m^6^A sites within *Tbx19* mRNA, we performed in silico prediction using the SRAMP web server (http://www.cuilab.cn/sramp). This analysis revealed four high‐confidence m^6^A modification sites at nucleotide positions 465, 500, 773 and 1201 (Figure 7C,D). Subsequent validation by methylated RNA immunoprecipitation coupled with quantitative PCR (MeRIP‐qPCR) revealed that, compared with the control group, *Ythdf3* overexpression (*Ythdf3*‐OE) significantly increased the m^6^A methylation level of *Tbx19* mRNA, specifically within the nucleotide regions encompassing positions 465–500 and 1201 (Figure 7E). These findings suggest that YTHDF3 may enhance *Tbx19* expression by regulating the stability of its transcripts in an m^6^A modification–dependent manner.

### 3.7 Overexpression of *Ythdf3* Enhanced the Osteogenic Capability of OP‐BMSCs in Vivo

To validate the regulatory role of YTHDF3 in the osteogenesis of BMSCs in vivo, we established a bilateral calvarial defect model in OP rats (Figure S2A). Initially, subsequent observation via fluorescence microscopy confirmed extensive colonisation of OP‐BMSCs within the BCP scaffolds (Figure S2B). Scanning electron microscopy (SEM) further revealed that OP‐BMSCs adhered abundantly to the surface of BCP (Figure S2C). These results indicate that OP‐BMSCs can successfully colonise and grow on BCP surfaces. Following this, BCP ceramics loaded with either control OP‐BMSCs or OP‐BMSCs overexpressing *Ythdf3* were implanted into the left and right calvarial defects, respectively. Samples were collected and analysed at 8 and 12 weeks post‐implantation.

At 12 weeks post‐transplantation, micro‐CT analysis revealed that the *Ythdf3* overexpression group exhibited a significant increase in bone volume fraction (BV/TV) and trabecular number (Tb.N), along with a marked reduction in trabecular separation (Tb.Sp). Although not statistically significant, samples at 8 weeks also showed an upward trend in BV/TV and Tb.N (Figure 8A,B). Similarly, H&E and Masson staining demonstrated a pronounced increase in new bone matrix formation in the *Ythdf3* overexpression group compared with the control group (Figure 8C). These findings suggest that *Ythdf3* overexpression enhances the osteogenic potential of BMSCs from OP rats, indicating that YTHDF3 can promote the osteogenic differentiation of BMSCs in vivo.

**FIGURE 8:**
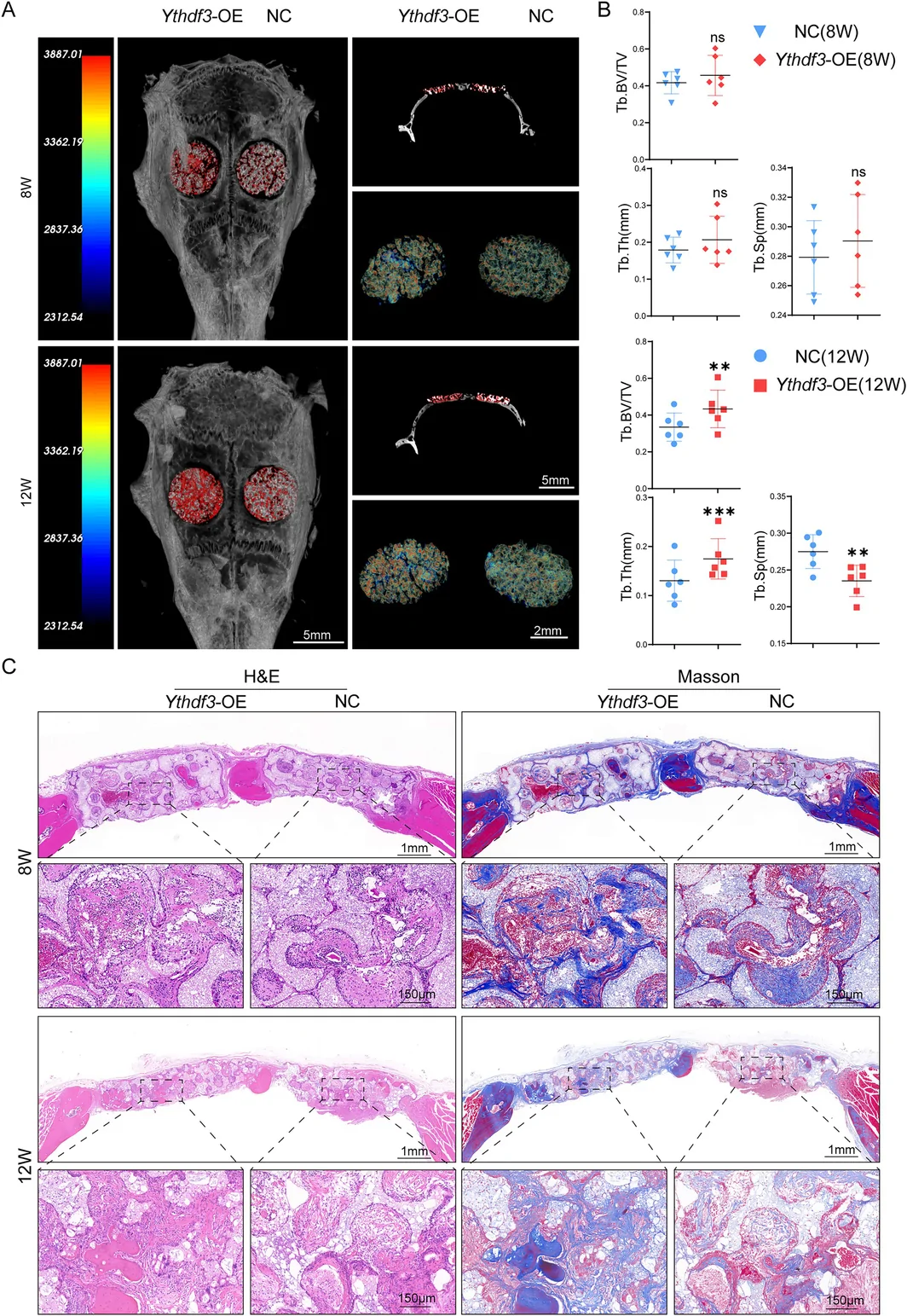
YTHDF3 promoted the osteogenic differentiation of BMSCs in vivo. (A) Representative micro‐CT images showing bilateral calvarial defects in rat calvaria at 8 and 12 weeks post‐BCP implantation. (B) Statistical analysis of bone parameters after BCP implantation into bilateral calvarial defects. (C) Representative images of H&E and Masson staining showing calvarial defects after BCP implantation. All data are presented as the means ± SD from six independent experiments and were analysed using a paired two‐tailed Student's *t*‐test (*n* = 6). **p < 0.01, ***p < 0.001, ns, not significant.

## 4 Discussion

OP is a systemic skeletal disorder characterised by reduced bone mass and microarchitectural deterioration of bone tissue, leading to enhanced bone fragility and fracture risk [33]. The pathophysiological basis of OP primarily involves an imbalance between bone formation and resorption, rooted in the diminished osteogenic differentiation and enhanced adipogenic propensity of BMSCs [34, 35, 36]. Here, we uncover a pivotal role for YTHDF3 in orchestrating the osteogenic differentiation of BMSCs and identify the transcription factor TBX19 as a potential downstream effector of YTHDF3. Our findings not only elucidate a novel YTHDF3‐TBX19 axis in regulating BMSCs' fate determination but also highlight potential therapeutic targets for stem cell‐based interventions in OP.

m^6^A, the most prevalent post‐transcriptional modification in eukaryotic mRNA, plays a pivotal role in regulating BMSCs differentiation [37, 38, 39]. Dot blot assay revealed that the overall m^6^A modification in BMSCs showed a decreasing trend after OP: the level of m^6^A methylation was significantly lower in OP‐BMSCs compared to the control group. This led us to hypothesise that enhancing m^6^A levels could potentiate the osteogenic capacity of OP‐BMSCs—a notion supported by prior studies [26, 40]. Existing research has largely focused on the dynamic m^6^A deposition and removal mediated by methyltransferases (e.g., the METTL3/METTL14 complex) and demethylases (e.g., FTO and ALKBH5) [41, 42, 43, 44]. However, the functional outcome of m^6^A modification is ultimately decoded by specific reader proteins that recognise m^6^A sites and dictate the fate of target mRNAs, influencing their splicing, nuclear export, stability, translation efficiency and decay. For instance, the nuclear reader YTHDC1 facilitates the export of m^6^A‐modified transcripts; within the cytoplasm, YTHDF2 and YTHDC2 often promote degradation of m^6^A‐marked mRNAs, while YTHDF1 is widely reported to promote translation in diverse biological contexts [18, 45, 46, 47]. YTHDF3 exhibits multifaceted functions: it not only collaborates with YTHDF1 and YTHDF2 to regulate mRNA translation and degradation, but also operates independently, with its roles demonstrating marked environmental and disease specificity.

Recent studies implicate YTHDF3 in processes ranging from oncogenesis and inflammation to stem cell differentiation [23]. For example, it regulates the autophagy process by promoting the translation of FOXO3 mRNA [48], or in AS, stabilises IL32 mRNA in an m^6^A‐dependent manner to promote aberrant osteogenesis [25]. Nevertheless, its precise role and downstream targets in OP remain poorly defined. Our study demonstrates that YTHDF3 is significantly downregulated in OP‐BMSCs. Functional assays confirmed that *Ythdf3* knockdown inhibited osteogenesis, while its overexpression enhanced differentiation, identifying it as a key positive regulator of BMSCs osteogenesis. Through RNA‐sequencing analyses, we further identified the transcription factor TBX19 as a downstream effector of YTHDF3.

Multiple members of the T‐box transcription factor family have been found to be associated with limb development and bone formation [49, 50, 51, 52]. TBX19, a member of the T‐box transcription factor family, is known for its critical role in pituitary development and cell fate determination [53, 54]. We observed that TBX19 was significantly upregulated following *Ythdf3* overexpression, whereas knockdown of *Tbx19* markedly impaired osteogenic differentiation. Notably, knockdown of *Tbx19* abolished the pro‐osteogenic effect of *Ythdf3* overexpression, indicating that TBX19 is an essential effector downstream of YTHDF3. Furthermore, we demonstrated through RIP‐qPCR, RNA stability assays and MeRIP‐qPCR that YTHDF3 enhances the stability of *Tbx19* mRNA in an m^6^A‐dependent manner, thereby upregulating *Tbx19* expression.

Our study is the first to identify TBX19—a transcription factor traditionally associated with pituitary development—as a critical downstream effector of YTHDF3 in osteogenic differentiation. We demonstrate that YTHDF3 promotes BMSC osteogenesis by upregulating TBX19, thereby revealing a previously unrecognised role for TBX19 beyond the endocrine system. Critically, OP‐BMSCs overexpressing YTHDF3 exhibit robust bone regeneration when seeded onto BCP scaffolds in vivo, underscoring the therapeutic potential of targeting the YTHDF3–TBX19 axis. While lentiviral‐mediated overexpression provides a powerful proof‐of‐concept platform, its clinical translation is limited by safety and regulatory concerns. Future efforts should therefore focus on developing clinically translatable approaches—such as small‐molecule agonists, peptide mimetics, or advanced nucleic acid–based delivery systems—to selectively enhance YTHDF3 activity in BMSCs [55, 56].

Despite these advances, the molecular mechanisms by which TBX19 governs BMSC fate remain poorly understood and warrant further investigation. Given the high sequence and structural conservation of both YTHDF3 and TBX19 between mice and humans, we hypothesise that this regulatory axis may also contribute to the pathogenesis of human OP. Direct validation in human bone tissues and hBMSCs derived from osteoporotic patients is thus an essential next step toward clinical relevance.

In summary, our study uncovers a previously unrecognised YTHDF3–TBX19 regulatory axis that critically governs osteogenic differentiation of BMSCs. We demonstrate that YTHDF3 enhances the stability of *Tbx19* mRNA in an m^6^A‐dependent manner, thereby upregulating TBX19 expression. Elevated TBX19, in turn, drives the transcriptional activation of key osteogenic master regulators, such as RUNX2 and OPN, ultimately promoting BMSC commitment to the osteoblast lineage. Using an ovariectomy‐induced osteoporotic rat model, we further validate that Ythdf3 potentiates the osteogenic capacity of BMSCs within the pathological bone microenvironment. Collectively, these findings establish the YTHDF3–TBX19 axis as a pivotal determinant of BMSC fate specification and reveal how an m^6^A reader–transcription factor cascade can precisely orchestrate cell differentiation programmes. Importantly, this work not only expands our understanding of post‐transcriptional regulation in skeletal biology but also highlights the YTHDF3–TBX19 pathway as a promising therapeutic target for OP‐related bone loss and regeneration.

## Author Contributions

Qianke Tao conducted the experiments, performed the animal and cell‐based assays, and drafted and revised the manuscript. Qiaonan Ye assisted with the animal and cell experiments and performed manuscript proofreading. Chengpeng Yang, Xuemei Long and Yuping Xie were responsible for model construction and assisted in the animal experiments. Long Bai, Zhiyuan Zhang and Qianwei Li carried out data acquisition and analysis, and participated in manuscript review and revision. Dan Tan and Jingang Xiao provided the overall research direction, financial support and critical guidance for the study. All authors reviewed and approved the final manuscript.

## Funding

This work was supported by National Natural Science Foundation of China (82370938), Science and Technology Department of Sichuan Province (2024NSFSC2087), Open Project of State Key Laboratory of Oral Diseases (SKLOD2025OF05), Science and Technology Project of Health Commission of Sichuan Province (24WXXT11), Luzhou Municipal Science and Technology Program Project (2025MYF002, 2025MYF024), Stomatological Project of Southwest Medical University (2025KQZX02, 2025KQZX09), Research Start‐up Fund for Doctoral Fellows of the Affiliated Stomatological Hospital of Southwest Medical University (2025BS04), Young Scientists Research Ascending Program of the Affiliated Stomatological Hospital of Southwest Medical University (2023KQ04), Scientific Research Project of Southwest Medical University (2025JC113) and Sichuan Medical Association Youth Innovation Project (Q20250047).

## Ethics Statement

The animal experiments performed were approved by the Laboratory Animal Center of Southwest Medical University (20230703‐016).

## Conflicts of Interest

The authors declare no conflicts of interest.

## Supporting information


**Figure S1:** Primary culture and Identification of BMSCs. (A) Typical spindle‐shaped adherent morphology of primarily cultured BMSCs (scale bar = 200 μm). (B) Flow cytometric analysis of surface markers (CD29, CD44, CD90, CD31, CD34, CD45) on primary BMSCs. (C) Histochemical staining for trilineage differentiation potential of BMSCs: osteogenesis (Alizarin Red S, ARS), adipogenesis (Oil Red O, ORO), and chondrogenesis (Alcian Blue, AB).

**Figure S2:** BCP/BMSCs construct for calvarial defect repair. (A) Schematic diagram of a bilateral critical‐size calvarial defect model (5 mm in diameter). (B) The status of BCP material with or without BMSCs co‐culture was observed under fluorescence microscopy after DAPI staining (scale bar = 100 μm). (C) The surface morphology of BCP material and the adhesion of BMSCs after co‐culture were analysed using scanning electron microscopy (SEM).

## Data Availability

The data that support the findings of this study are available from the corresponding author upon reasonable request.
