# Supplementary figures and images for: YTHDF3 Enhances Osteogenic Differentiation of Bone Marrow Mesenchymal Stem Cells in Osteoporosis by Promoting TBX19 Expression
Source: Cell Prolif. 2026 Jun 3:e70240. Online ahead of print. doi: 10.1111/cpr.70240 (PMC13326000; doi:10.1111/cpr.70240)

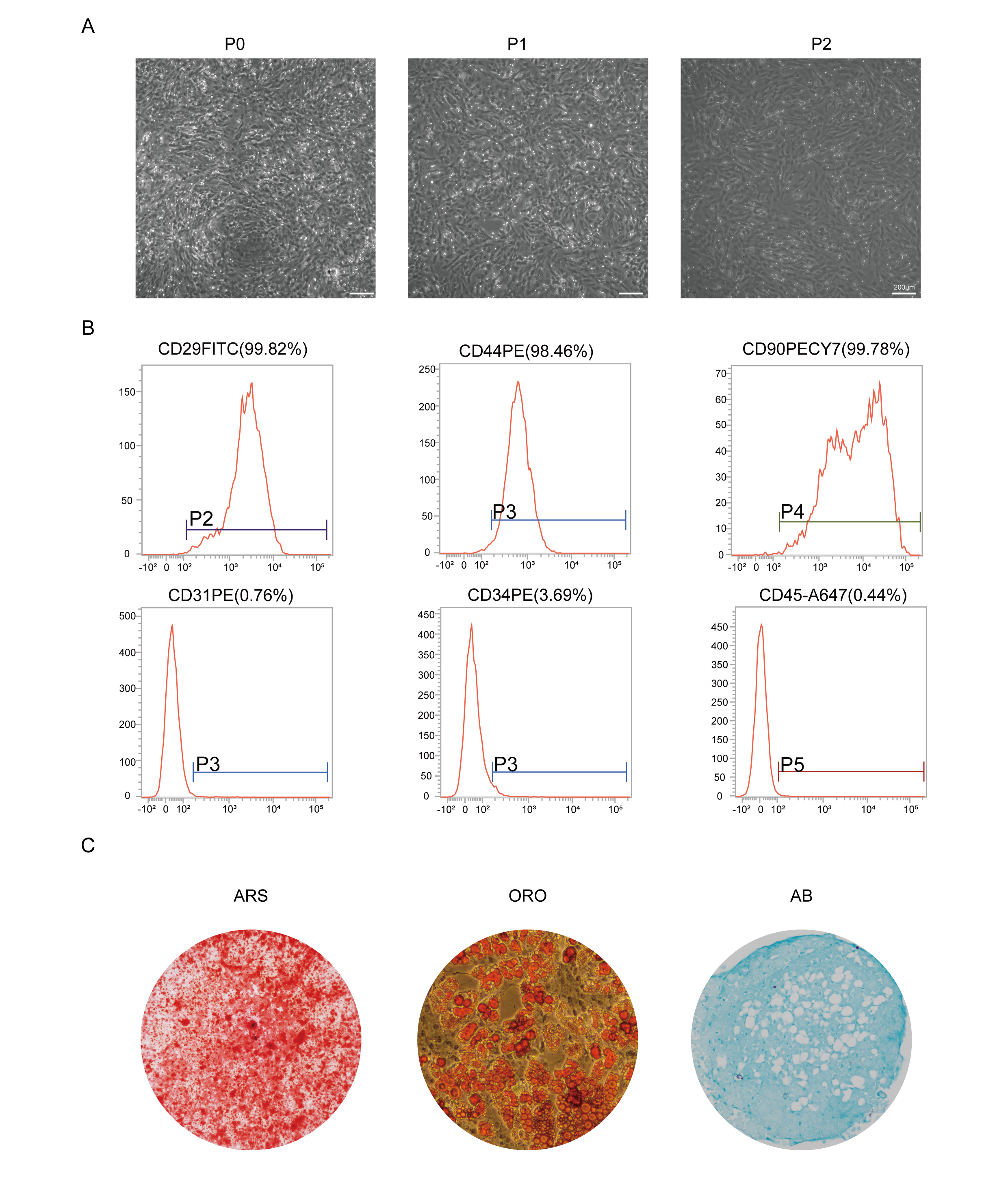

Supplement: Supplementary file 1 — Figure S1: Primary culture and Identification of BMSCs. (A) Typical spindle‐shaped adherent morphology of primarily cultured BMSCs (scale bar = 200 μm). (B) Flow cytometric analysis of surface markers (CD29, CD44, CD90, CD31, CD34, CD45) on primary BMSCs. (C) Histochemical staining for trilineage differentiation potential of BMSCs: osteogenesis (Alizarin Red S, ARS), adipogenesis (Oil Red O, ORO), and chondrogenesis (Alcian Blue, AB). [file CPR-9999-e70240-s002.png]

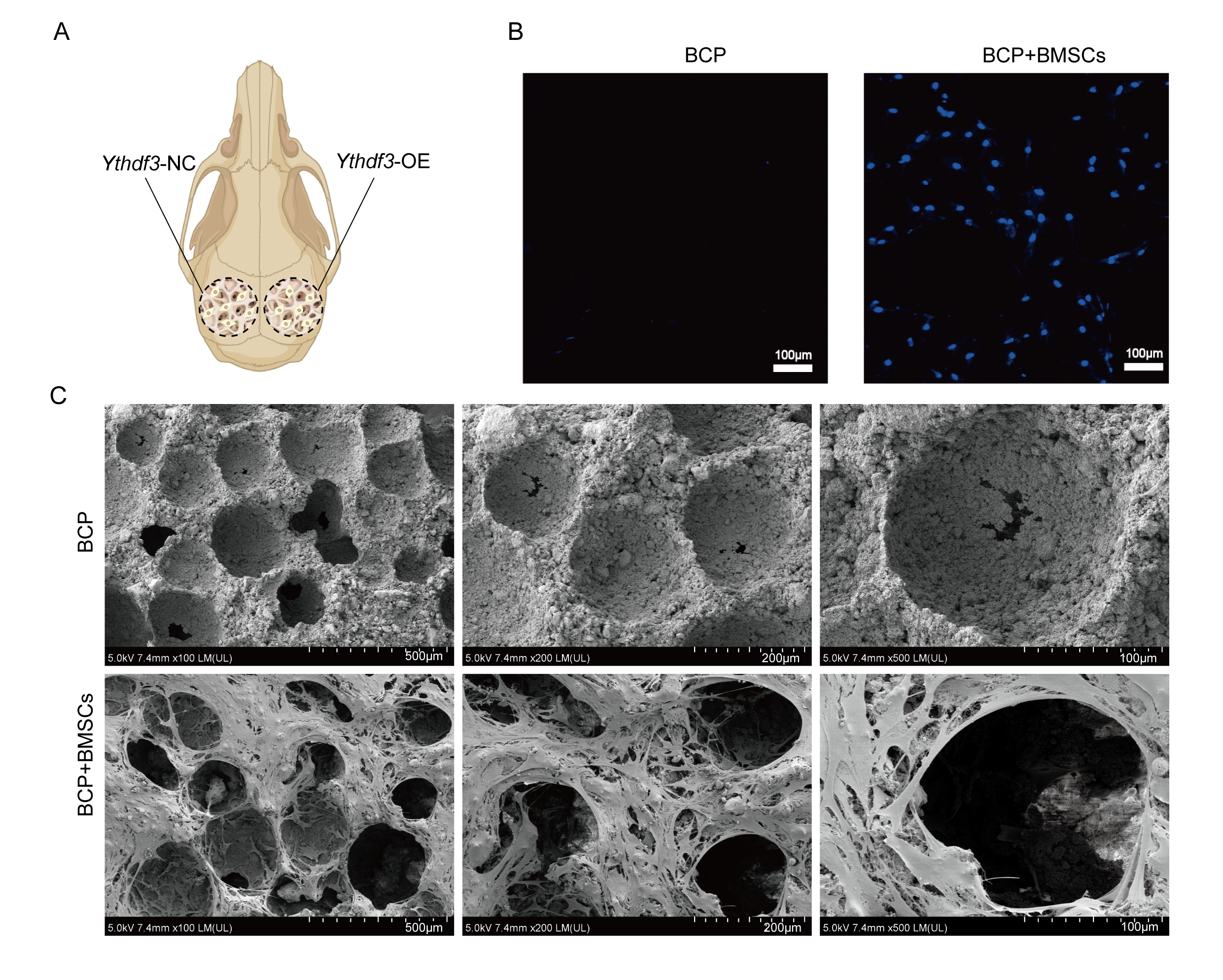

Supplement: Supplementary file 2 — Figure S2: BCP/BMSCs construct for calvarial defect repair. (A) Schematic diagram of a bilateral critical‐size calvarial defect model (5 mm in diameter). (B) The status of BCP material with or without BMSCs co‐culture was observed under fluorescence microscopy after DAPI staining (scale bar = 100 μm). (C) The surface morphology of BCP material and the adhesion of BMSCs after co‐culture were analysed using scanning electron microscopy (SEM). [file CPR-9999-e70240-s001.png]
